# Translation, Cross-Cultural Adaptation and Validation of the Myofascial Adhesions for Patients after Breast Cancer (MAP-BC) Evaluation Tool: Spanish Version

**DOI:** 10.3390/ijerph19074337

**Published:** 2022-04-04

**Authors:** María Jesús Casuso-Holgado, Beatriz Ostos-Díaz, María Jesús Muñoz-Fernández

**Affiliations:** 1 Department of Physiotherapy, Faculty of Nursing, Physiotherapy and Podiatry, Universidad de Sevilla, C/ Avicena s/n, 41009 Seville, Spain; 2 Department of Physiotherapy, University School Francisco Maldonado, Avd. de los Cipreses s/n, 41640 Osuna, Spain; mariajmf@euosuna.org

**Keywords:** cross-cultural adaptation, MAP-BC, breast cancer, cicatrix

## Abstract

(1) Background: The Myofascial Adhesions for Patients after Breast Cancer (MAP-BC) evaluation tool is a quantitative measure for the evaluation of tissue adhesions in breast cancer patients. The aims of this study were to create a Spanish version of the MAP-BC and to test its convergent validity and responsiveness. (2) Methods: Translation and cross-cultural adaptation were performed in five phases according to international guidelines. For the analysis of the convergent validity and responsiveness, a sample of 77 patients after breast cancer surgery were analysed at two timepoints (T1: immediately after the stitches were taken out; T2: one month after surgery). The COSMIN (Consensus-based Standards for the selection of health status Measurement Instruments) guidelines and checklist were used to verify the whole adaptation and validation process. (3) Results: Translation and cross-cultural adaptation of the original English version resulted in an easily understandable Spanish version of the tool. A moderate convergent validity (*r* = 0.438) with the Patient and Observer Scar Assessment (POSAS) at T1 and a strong correlation at T2 (*r* = 0.816) were observed. A moderate standardised response mean (0.45) and effect size (0.63) were also observed. (4) Conclusions: The MAP-BC Spanish version is a useful tool that could be incorporated into the evaluation of scars and surrounding tissues in breast cancer patients. It has shown a moderate convergent validity and responsiveness.

## 1. Introduction

Breast cancer is the most commonly diagnosed type of cancer among women, with around 2.1 million cases being diagnosed every year [1]. In Spain, the incidence of this disease is increasing, and it is estimated that one in eight Spanish women will be diagnosed with breast cancer in her lifetime [2]. Luckily, although the incidence rate is increasing, the mortality rate is falling, thanks to advances in early detection and a variety of medical treatments, such as surgery [1,3].

Nowadays, breast cancer surgery techniques tend to be less invasive. However, even breast-conserving surgery can have physical consequences [4]. Surgical interventions may cause side effects, such as scar adhesions, breast deformity, due to removal of some of the volume of the mammary gland, and postoperative fibrosis and scar formation [5]. Tissue complications following radiotherapy are also inevitable [6]. Adhesions in the scars and surrounding tissues, due to surgery or radiotherapy, are rigid bands of fibrous tissue, preventing the displacement, and movement, between different layers of tissues, resulting in limited mobility [7,8].

Surgical scars have a negative impact on the quality of life of patients operated on for breast cancer. Recently, Gass et al. (2019) [3] performed a national survey in the United States, showing that 85.7% of women who underwent a simple mastectomy felt emotionally affected, and had decided not to wear certain kinds of clothing. In addition, 67% of these women did not like the location of their scar. Upper limb dysfunction after breast cancer surgery and radiotherapy can arise as a consequence of myofascial dysfunction [8,9,10]. Early treatment of scars, adhesions and tissues with surrounding fibrosis can help to avoid these adhesions and restrictions in the connective tissue becoming definitively established, and causing a decrease of functionality [11]. For this reason, it is important to have a tool that measures the degree of myofascial/tissue adhesions in scars after breast cancer to aid early diagnosis.

De Groef et al. (2017) [12] created a tool to assess myofascial/tissue adhesions in patients after breast cancer named the Myofascial Adhesions for Patients after Breast Cancer (MAP-BC) evaluation tool, but this tool has not been adapted for Spanish evaluators. This instrument is a quantitative measure used for the subjective assessment of adhesions by palpation. It is very visual, since it has graphical representations, and is brief, which allows for quick evaluation of patients. It has also been shown to be a valid and reliable tool for the correct diagnosis of the state of myofascial tissue after breast cancer [12,13].

Currently, there is no assessment tool in Spanish for myofascial adhesions in patients after surgical and adjuvant treatments of breast cancer. Therefore, it would be useful to have a Spanish version of the MAP-BC evaluation tool, allowing Spanish-speaking physical therapists to perform early detection of myofascial/tissue adhesions in breast cancer patients.

Taking into account the above statements, the aims of this study were to create a translated version of the MAP-BC for the Spanish population, and to test its convergent validity and responsiveness in breast cancer patients undergoing breast surgery.

## 2. Materials and Methods

### 2.1. Study Design

This is a cross-cultural adaptation and validation study of the MAP-BC Spanish version. This study is part of a broader trial concerning early physiotherapy after breast cancer surgery (ACTRN12618000719235). Before conducting the study, ethical approval for the specific aim of this research was obtained from the Local Ethics Committee on Human Research (PEIBA nº 1781-N-18, on 6 December 2019).

Informed consent was signed by all participants prior to surgical intervention, following the recommendations of the Declaration of Helsinki, and the legal regulations of Spain regarding clinical research (in particular, Law 14/2007 on biomedical research). The patients were also sufficiently informed, in a clear and precise way, of all aspects of the study.

This study was organised in two steps. First, the English version of the MAP-BC was translated and cross-culturally adapted for the Spanish population. Second, its convergent validity and responsiveness was assessed in a prospective study. The COSMIN (Consensus-based Standards for the selection of health status Measurement Instruments) guidelines and checklist were used to verify the whole adaptation and validation process [14,15].

### 2.2. Development of the Spanish Version of the MAP-BC: Translation and Cross-Cultural Adaptation

Translation and cross-cultural adaptation was performed in the following five phases according to international recommendations [16,17].

#### 2.2.1. Preparation

One of the researchers (MJMF) contacted the MAP-BC developers (Prof. An De Groef) and obtained permission to use and translate this tool into Spanish.

#### 2.2.2. Forward Translation and Reconciliation

The MAP-BC original version was translated into Spanish by two independent bilingual translators (native Spanish language speakers). Then, both translators met to form a consensus and a single version was achieved.

#### 2.2.3. Backward Translation

A native English-speaking translator, unaware of the original version of the MAP-BC, back-translated the questionnaire into English.

#### 2.2.4. Review and Harmonisation

A committee, composed of the translators and five expert physical therapists in the field, reviewed the translated and original versions of the MAP-BC. They reviewed the idiomatic, semantic and conceptual equivalences, and other elements, such as titles and instructions. Finally, the committee agreed on the final version of the Spanish MAP-BC tool.

#### 2.2.5. Pilot Testing

Two physical therapists from the research team (MJMF, BOD) examined a pilot sample of 15 patients independently [12]. Experiences, difficulties and findings were discussed afterwards.

### 2.3. Assessment of Convergent Validity and Responsiveness

Convergent validity of the Spanish version of the MAP-BC was evaluated by analysing the correlations between this tool and the observer subscale from the Patient and Observer Scar Assessment (POSAS) [18,19]. For this purpose, a sample of patients, who had undergone breast cancer surgery and adjuvant therapy, was analysed. Inclusion criteria were as follows: women aged 18–90 years, clinically diagnosed with breast cancer and undergoing breast cancer surgery. Exclusion criteria were: refusal to participate, or the existence of any medical condition that may suppose a contraindication.

For responsiveness analysis, participants were evaluated twice after surgery: immediately after the stitches were taken out (T1) and one month after surgery (T2). Between T1 and T2 participants received two group sessions of scar recovery (scar massage and stretching). Adjuvant therapy was not received between evaluations. All evaluations were carried out by two experienced physical therapists.

#### 2.3.1. POSAS

This assessment is composed of two separate scales (the observer and the patient scales) [18,19]. The observer scar assessment scale rates five variables: vascularity, pigmentation, thickness, surface roughness and pliability. Each variable uses a 10-point scoring system, with 1 representing normal skin. For pigmentation, there is an additional ranked subscale for pigment type: hyperpigmentation, mixed, or hypopigmentation. Ratings of individual variables may be summed to obtain a total score ranging from 5 to 50, with 5 representing normal skin. This assessment was validated for the evaluation of scars after breast cancer surgery by Truong et al. (2007) [19]. For this research, the Spanish version of the POSAS was used [20].

#### 2.3.2. MAP-BC

This tool was developed and validated by De Groef et al. (2017, 2018) [12,13] to quantitatively evaluate tissue/myofascial adhesions in scars after breast cancer treatments. The degree of adhesion is scored at three levels of depth (skin, superficial and deep) and, in turn, on a scale of four points in each area (between 0: no adhesion and 3: very strong adhesion). The areas evaluated are as follows: axillary scar, breast scar/mastectomy scar, pectoralis region, frontal chest wall, lateral chest wall, axilla and inframammary fold. The final score is obtained from the sum of the three levels of each area; the minimum score is 0 and the maximum score is 63.

### 2.4. Statistical Analysis

Data analysis was carried out using the Statistical Package for the Social Sciences (SPSS, version 20.0, IBM Corp., Armonk, NY, USA). Normal data distribution was tested by the Shapiro-Wilk test. For convergent validity, the strength of Pearson correlations was interpreted as low (<0.25), fair (0.25–0.50), moderate to good (0.50–0.75) and good to excellent (>0.75) [21]. Responsiveness was analysed using a *t*-test for paired samples. The standardised response mean (SRM = mean change/standard deviation change) and effect size (ES = mean change/basal standard deviation) were calculated. For both statistics, responsiveness was considered to be low (0.20), moderate (0.50) or high (0.80) [22,23] For all analyses, a statistical significance level of 95% (*p* < 0.05) was adopted.

## 3. Results

### 3.1. Development of the Spanish Version of the MAP-BC: Translation and Cross-Cultural Adaptation

In order to achieve a high-quality cross-cultural adaptation of the MAP-BC, some conceptual and idiomatic changes were made after the expert committee meeting. The phrase “The tool” in the title was replaced with “Tool”; the word “physiotherapists” was changed to “therapists” in the instructions; the word “adhesion” was changed to the word “adherence”; and the word “moves” was changed to the word “slide”. The full final version of the Spanish MAP-BC can be found in Appendix A. No difficulties were found after pilot testing of the final version.

### 3.2. Assessment of Convergent Validity and Responsiveness

**Figure 1:**
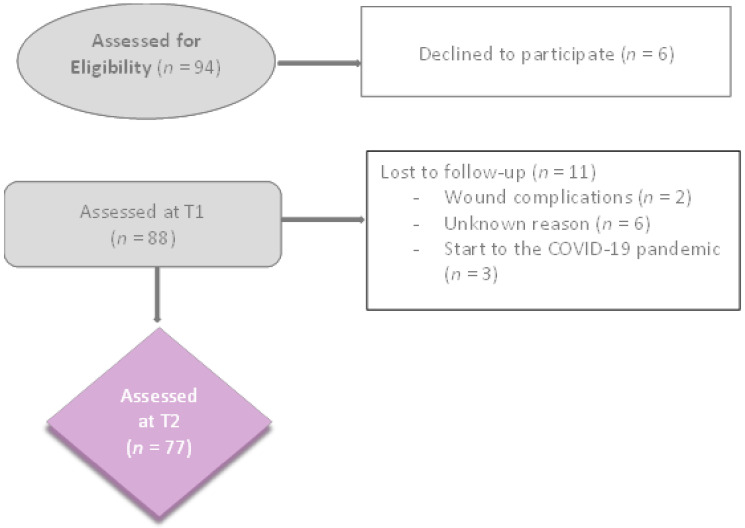
Flow chart of the participants.

A total of 94 patients were selected for the study. Of these, six patients declined to participate in the study and 77 completed the two evaluations. After evaluation at T1, two women refused to continue, due to wound complications or fear of scar manipulation, six women dropped out, for unknown reasons, and three women were unable to complete the assessment, due to the onset of the COVID-19 pandemic (Figure 1).

The mean age of the sample was 59.57 years (±11.93). The majority of subjects (77.9%) underwent breast-conserving surgery, 22% had a simple mastectomy and 24.7% had received adjuvant therapy before surgery (radiotherapy, 2.6%; chemotherapy, 20.8%).

Regarding surgical scar assessments, the mean of the POSAS Scale at T1 was 24.81 (±10.68) and at T2 16.81 (±12.60). On the other hand, the mean of the MAP-BC Scale at T1 was 24.5 (±11.54) and at T2 17.16 (±17.03). A detailed description of the sample and evaluations is shown in Table 1.

**Figure 2:**
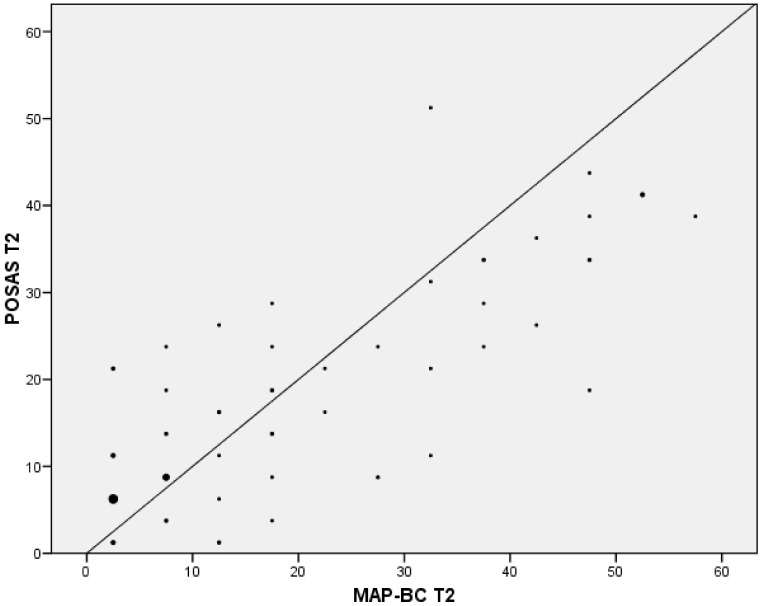
Correlation at T2 between the Myofascial Adhesions for Patients after Breast Cancer (MAP-BC) Spanish version with the Patient and Observer Scar Assessment (POSAS).

An overall strong and significant correlation was found between MAP-BC and POSAS scales at T2, with a Pearson’s coefficient of 0.816 (*p* < 0.001) (Figure 2). However, the correlation at the earliest evaluation (T1) was found to be moderate, with a Pearson´s coefficient of 0.438 (*p* < 0.001). The t-test revealed a significant difference between T1 and T2 scores (*p* < 0.001). Moreover, a moderate SRM (0.45) and ES (0.63) were observed (Table 2).

## 4. Discussion

The aim of this study was to create the Spanish version of the MAP-BC evaluation tool and to test its concurrent validity and responsiveness for the assessment of tissue adhesions after breast cancer treatments.

### 4.1. Development of the Spanish Version of the MAP-BC: Translation and Cross-Cultural Adaptation

Translation and cross-cultural adaptation of the original English version resulted in an easily understandable Spanish version of this tool. Some moderate idiomatic changes were needed, but no difficulties were found after the pilot testing of the tool. This result suggests that the Spanish version of the MAP-BC can report good face and content validity in accordance with the original scale [13], although this question needs to be tested further in the future.

Few studies have validated instruments for the evaluation of scars for Spanish speakers. To our knowledge, among the most frequently used scales for this purpose (Vancouver Scar Scale, Visual Analogue Scale, Manchester Scar Scale and POSAS) [24,25], only a Spanish version of the POSAS scale has been transculturally adapted [20]. A Spanish version of the Patient Scar Assessment Questionnaire (PSAQ) for neck and head surgery has also been recently developed [26]. The development of the Spanish version of the MAP-BC could help Spanish physicians and physiotherapists, not only in the evaluation of scars, but also in the evaluation of tissue adhesions after breast cancer.

### 4.2. Convergent Validity and Responsiveness

Convergent validity between the MAP-BC and POSAS was moderate at the short-term evaluation, and strong when comparisons were made one month after surgery. To our knowledge, a gold-standard evaluation for myofascial adhesions does not exist. Thus, although the POSAS scale evaluates other aspects of scars, such as pigmentation and vascularity, which may have influenced the between-measures correlations, it also focuses on thickness, surface roughness and pliability of the scar tissues. As only two participants have received radiotherapy, we hypothesised that tissue adhesions need a delay after surgery. Therefore, the clinical use of the MAP-BC would be limited in the short-term in patients without adjuvant radiotherapy, but would be useful for longer- term evaluation.

The concurrent validity of the original version of the MAP-BC has also been tested previously. However, for this purpose, a cutometer was used, an objective instrument that measures the vertical deformation of the skin in millimetres when the skin is pulled, instead of as a subjective scale, as in our study. Similarly to our findings, the authors found a moderate concurrent validity of the MAP-BC evaluation tool for the mastectomy scar itself, and suggested that other tools may be better suited to explore the concurrent validity of the MAP-BC [13].

A significant difference between measures over time was observed, with a moderate SRM and ES. We hypothesise that the short period between evaluations could explain these modest results. Responsiveness of the original MAP-BC has not been previously analysed, making it impossible to discuss our findings [12,13]. For this purpose, future research should include a larger number of subjects undergoing radiotherapy, and a longer evaluation framework.

The field of scar assessment lacks standardised methodology, with different instruments used in the clinical setting [27]. Particularly for breast surgery scars, an integrated method, based on the Vancouver Scar Scale, the McGill Pain Questionnaire and the patients’ perspectives, has been reported to be feasible for the evaluation of these type of scars [28].

The Spanish MAP-BC proved to be acceptable, easily understood and could be administered in about 15 min. Taking into account our results, the adapted version of the MAP-BC could foster further evaluation of tissue adhesions by Spanish evaluators in patients with breast cancer.

This study has some limitations. First, the convergent validity of the MAP-BC was tested using another subjective tool. Although a gold-standard measure does not exist for the evaluation of scar adhesions, the use of an objective measure would be of interest. Second, correlations between change scores, or the area under the Receiver Operator Curve (ROC), w not calculated. Third, intra- and inter-rater reliability was not analysed. Intra-rater reliability is expected to be good [12], but it would be useful to know the concordance between evaluators of the Spanish version of the MAP-BC. Finally, pilot testing of the tool was conducted, but face and content validity were not analysed.

## 5. Conclusions

The original MAP-BC evaluation tool was successfully translated and transculturally adapted into Spanish. The Spanish MAP-BC version showed itself to be a useful and valid tool to assess surgical scars and surrounding tissue adhesions after breast cancer. Concurrent validity was particularly strong one month after surgery and its responsiveness was moderate.

To the best of our knowledge, this is the first study adapting and validating the MAP-BC evaluation tool into Spanish. Future research should focus on testing its reliability, and content and face validity.

## Figures and Tables

**Table 1:** Characteristics of the participants and evaluations of surgical scars.

Demographics and Characteristics	Number of Subjects = 77(%)
**Age at Diagnosis, years**	Mean ± SD: 59.51 ± 11.93 Minimum: 31 Maximum: 85
<45	8 (10.38)
45–54	22 (28.57)
55–64	15 (19.48)
+65	32 (41.55)
**Body Mass Index, Baseline, kg/m^2^**	Mean ± SD: 31.5 ± 4.94 Minimum: 17.97 Maximum: 49.33
Normal: 18.5–24.9	28 (36.8)
Overweight: 25–29.9	30 (38.9)
Obese: ≥30	18 (23.7)
**Ethnicity**	
Caucasian	74 (96.1)
African	1 (1.3)
Latin American	2 (2.6)
**Type of Breast Cancer**	
Ductal Carcinoma in situ (DCIS)	19 (24.6)
Invasive Ductal Carcinoma (IDC)	54 (70.1)
Lobular Carcinoma in situ	3 (3.9)
Invasive Lobular Carcinoma	1 (1.3)
**Stage of Breast Cancer**	
0	7 (9.7)
IA	44 (61.1)
IIA	11 (14.3)
IIIA	1 (1.3)
IB	3 (4.1)
IIB	6 (8.3)
**Type of breast surgery**	
Simple unilateral mastectomy	17 (22.0)
Breast conserving surgery	60 (77.9)
**Numbers of lymph nodes removed**	Mean ± SD: 2.01 ± 1.12 Minimum: 1 Maximum:6
**Positive Lymph Nodes**	
Yes	11 (14)
No	66 (86)
**Side Involved**	
Right	42 (54.5)
Left	34 (44.1)
Bilateral	1 (1.2)
**Involved side to hand dominance**	
Ipsilateral side	43 (55.8)
Contralateral side	34 (44.1)
**Adjuvant Therapy**	
Yes	19 (24.7)
No	58 (75.3)
**Type of Adjuvant Therapy**	
Radiation	2 (2.6)
Chemotherapy	16 (20.8)
POSAS (T1)	Mean ± SD: 24.81 ± 10.68
POSAS (T2)	Mean ± SD: 16.87 ± 12.60
MAP-BC (T1)	Mean ± SD: 24.50 ± 11.54
MAP-BC (T2)	Mean ± SD: 17.16 ± 17.03

MAP-BC: Myofascial Adhesions for Patients after Breast Cancer evaluation tool; POSAS: Patient and Observer Scar Assessment; T1: evaluation after removal of stitches; T2: evaluation one month after surgery.

**Table 2:** Validity and responsiveness of the MAP-BC Spanish version.

MAP-BC	Mean (SD)	*p*-Value	SRM	ES	Correlations between the MAP-BC and POSAS Scales
T1 evaluation	24.50 (11.54)				T1 evaluation: *r* = 0.438 (*p* < 0.001)
T2 evaluation	17.16 (17.03)	0.000	0.45	0.63	T2 evaluation: *r* = 0.816 (*p* < 0.001)

ES: effect size; MAP-BC: Myofascial Adhesions for Patients after Breast Cancer evaluation tool; POSAS: Patient and Observer Scar Assessment; SD: standard deviation; SRM: standardized response mean; T1: evaluation after removal of stitches; T2: evaluation one month after surgery.

## Data Availability

The data presented in this study are available on request, from the corresponding authors.

## Appendix A. Final MAP-BC Evaluation Tool: Spanish Version

Herramienta de evaluación para Adherencias Miofasciales en Pacientes que han sufrido Cáncer de Mama (herramienta de evaluación MAP-BC).

### Appendix A.1. Instrucciones para los Terapeutas:

Salvo que no pueda, el paciente debe colocarse en decúbito supino con la cabeza apoyada en una almohada y los brazos separados del cuerpo. El terapeuta se coloca en el lado homolateral. Se palpan los tres planos tisulares descritos más abajo. El grado de adherencia se sitúa entre 0 y 3 en cada nivel. La puntuación total se calcula como la suma de las puntuaciones de los diferentes niveles.

**Nivel**	**Instrucciones para la Palpación**
Piel	Sin presión vertical, deslizar la piel en todas las direcciones relativas a las estructuras anatómicas del nivel miofascial superficial.
Superficial	Deslizar la piel y los 6 tejidos subcutáneos del nivel superficial en todas las direcciones relativas al nivel miofascial profundo subyacente.
Profundo	Deslizar los tejidos en todas las direcciones relativas a las estructuras óseas subyacentes.

### Appendix A.2. Sistema de puntuación

**Puntuación**	**Grado de Deslizamiento del Tejido Restringido**
0	No hay restricción en el deslizamiento del tejido.
1	Restricción limitada que se libera inmediatamente.
2	Grave restricción en el deslizamiento del tejido.
3	El deslizamiento del tejido es imposible.

**1. CICATRIZ AXILAR *(90º Abducción)***	**2a. CICATRIZ DEL PECHO *(Cirugía Conservadora del Pecho)***	**2b. CICATRIZ DE MASTECTOMÍA *(Mastectomía)***	**3. REGIÓN DEL MÚSCULO PECTORAL *(90º Abducción)***

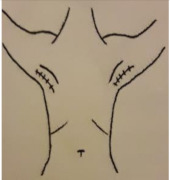
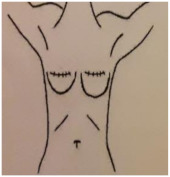
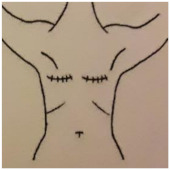
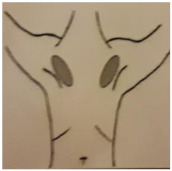
			
Piel: 0–1–2–3 Superficial: 0–1–2–3 Profundo: 0–1–2–3 **PUNTUACIÓN TOTAL:**	Piel: 0–1–2–3 Superficial: 0–1–2–3 Profundo: 0–1–2–3 **PUNTUACIÓN TOTAL:**	Piel: 0–1–2–3 Superficial: 0–1–2–3 Profundo: 0–1–2–3 **PUNTUACIÓN TOTAL:**	Piel: 0–1–2–3 Superficial: 0–1–2–3 Profundo: 0–1–2–3 **PUNTUACIÓN TOTAL:**
**4. PARED TORÁCICA FRONTAL**	**5. PARED TORÁCICA** **LATERAL**	**6. AXILA** *(90º Abducción)*	**7. PLIEGUE INFRAMAMARIO**

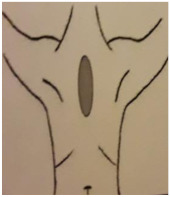
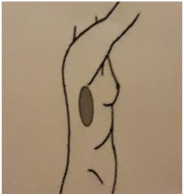
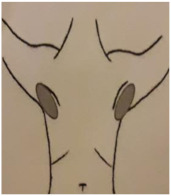
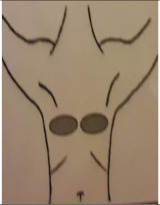
			
Piel: 0–1–2–3 Superficial: 0–1–2–3 Profundo: 0–1–2–3 **PUNTUACIÓN TOTAL:**	Piel: 0–1–2–3 Superficial: 0–1–2–3 Profundo: 0–1–2–3 **PUNTUACIÓN TOTAL:**	Piel: 0–1–2–3 Superficial: 0–1–2–3 Profundo: 0–1–2–3 **PUNTUACIÓN TOTAL:**	Piel: 0–1–2–3 Superficial: 0–1–2–3 Profundo: 0–1–2–3 **PUNTUACIÓN TOTAL:**
